# Antithrombotic Effect and Mechanism of Radix Paeoniae Rubra

**DOI:** 10.1155/2017/9475074

**Published:** 2017-02-16

**Authors:** Pingyao Xie, Lili Cui, Yuan Shan, Wen-yi Kang

**Affiliations:** ^1^Institute of Chinese Materia Medica, Henan University, Kaifeng 475004, China; ^2^Kaifeng Key Laboratory of Functional Components in Health Food, Kaifeng 475004, China

## Abstract

The compounds of Radix Paeoniae Rubra (RPR) were isolated and identified by bioassay-guided method, and antithrombotic effects and mechanism were investigated by the acute blood stasis rat model. The RPR extract was evaluated by APTT, TT, PT, and FIB assays in vitro. Results indicated that RPR extract exhibited the anticoagulant activity. In order to find active compounds, six compounds were isolated and identified, and four compounds, paeoniflorin (Pae), pentagalloylglucose (Pen), albiflorin (Ali), and protocatechuic acid (Pro), exhibited the anticoagulant activity in vitro. Therefore, the antithrombosis effects of RPR extract and four active compounds were investigated in vivo by measuring whole blood viscosity (WBV), plasma viscosity (PV), APTT, PT, TT, and FIB. Meanwhile, the levels of TXB_2_, 6-Keto-PGF_1*α*_, eNOS, and ET-1 were detected. Results suggested that RPR extract and four active compounds had the inhibition effect on thrombus formation, and the antithrombotic effects were associated with the regulation of vascular endothelium active substance, activating blood flow and anticoagulation effect.

## 1. Introduction

Thrombosis, the formation or presence of the thrombus in a blood vessel, is a multifactorial disease induced by promoting a combination of stasis and hypercoagulability [1]. Thrombotic diseases, especially heart disease and cerebrovascular thrombosis, have become primary causes of death and their incidence has been increasing each year [2]. It is well known that thrombosis is closely related to activated platelet adhesion, aggregation, secretion functions, and activation of intrinsic and extrinsic coagulation systems, which cause blood coagulation and fibrin formation. Although they have many causes [3], accumulation of thrombus is a key factor in the final analysis.

Many traditional Chinese herbal medicines have been used for thousands of years in clinical practice because of their proven efficacy, wide indications, high safety profile, and low toxicity [4]. Then Traditional Chinese Medicine (TCM) plays an important role in the clinic treatment of blood stasis in China, one of which is the Radix Paeoniae Rubra (RPR), which is the dried root of* Paeonia lactiflora* Pall. and* P. veitchii* Lynch [5]. It had the function of removing pathogenic heat from the blood and treating blood stasis and relieving pain [6, 7].

In Traditional Chinese Medicine, thrombotic disorders are described as blood stasis syndrome [8]. Though aspirin has been widely used as antithrombotic medicine in clinical practice, an increasing number of studies have indicated that some patients subpopulations do not respond to the antithrombotic effects of aspirin and may exhibit a degree of aspirin resistance. Therefore, interest in TCM from natural sources as a feasible alternative therapeutic agent for the prevention of blood stasis disorder in patients with “aspirin resistance” is growing [9].

RPR has some effects on activating blood circulation to dissipate blood stasis; however, the mechanism has been poorly studied. In this paper, antithrombotic effects and mechanism of extracts and the compounds from the RPR root were investigated.

## 2. Materials and Methods

### 2.1. Plant Materials

The RPR was purchased from Lerentang Pharmacy, Kaifeng, Henan Province, China, and a voucher specimen was identified by Professor Chang-qin Li of Henan University and deposited in the Herbarium of the Institute of Natural Products, Henan University.

### 2.2. Animals

Male and female Sprague-Dawley (SD) rats, weighing from 220 to 300 g, and male New Zealand white rabbits from 2.0 to 2.5 kg were obtained from the Experimental Animal Center of Henan Province (Zhengzhou, Henan, China) (12 h light/dark cycle, 25°C, and humidity 45 to 65%) and were fed with standard rodent diet and water ad libitum. The animal procedures were approved by the ethical committee in accordance with “Institute Ethical Committee Guidelines” for Animal Experimentation and Care. Animals were housed in standard cage.

### 2.3. Chemicals

 The following was used: PT, APTT, TT, and FIB Assay Kits (Shanghai Sun Biotech Co., Ltd.); Bovine Fibrinogen (Sigma, USA); Thrombin (Sigma, USA,); Urokinase (Biotechnology Co., Ltd., Hangzhou, Macao, and Asia); Agarose (GENE Company); Complex Tablet (Hutchison Whampoa Guangzhou Baiyunshan Chinese Medicine Company Limited, China); Rat Endothelin 1 (ET-1) Elisa Kit; Rat Endothelial Nitric Oxide Synthase (eNOS) Elisa Kit; Rat 6-Keto-PGF1*α* Elisa Kit; Rat Thromboxane B_2_ (TXB_2_) Elisa Kit (Nanjing Sengbiejia Biotech Co., Ltd.).

### 2.4. Extraction and Isolation

Air-dried and powdered roots of RPR (1.9 kg) were extracted with 70% ethanol at 60°C for two times (2 h each time) and then pressure was reduced to yield a viscous gummy material (570 g). The total extract was dissolved in 500 mL of water and then extracted with petroleum ether, ethyl acetate, and* n*-butanol. The PE, EtOAc, and BuOH extracts were concentrated under reduced pressure to give 6 g, 29 g, and 81.3 g, respectively. EtOAc extract (29 g) was subjected to silica gel H column chromatography and eluted with dichloromethane-ethyl acetate (100 : 0-0 : 100, v/v) to yield three fractions. The first fraction (4.6 g) was further separated on a silica gel column repeatedly to yield compound** 1** (123.1 mg). The second fraction (2.3 g) was purified by various column chromatography including silica gel and sephadex LH-20 to yield compound** 2** (53.5 mg) and compound** 3** (36.5 mg).* n*-Butanol extract (81.3 g) was purified on MPLC and then further separated on silica gel H and sephadex LH-20 column chromatography to get compounds** 4** (31.2 mg),** 5** (82.9 mg), and** 6** (38.4 mg), respectively. The six compounds were identified as paeoniflorin (**1**, Pae), pentagalloylglucose (**2**, Pen), benzoyl paeoniflorin (**3**, Ben), oxypaeoniflora (**4**, Oxy), albiflorin (**5**, Ali), and protocatechuic acid (**6**, Pro) by ^1^H NMR and ^13^C NMR. All the compounds were performed on HPLC, and the purity was over 98%.

### 2.5. Coagulation Time Test In Vitro

Blood samples were drawn from rabbit's auricular vein. After collection, the blood was decalcified by sodium citrate to prevent blood clotting, and then serums were separated from the plasma by centrifugation of 3,000 rpm at 5°C for 15 min [10]. APTT and PT were determined according to the literature [11]. In brief, serum (50 *μ*L) was mixed with 25 *μ*L of samples, next APTT assay reagent (50 *μ*L) was added and incubated for 5 min at 37°C, and then 25 mM CaCl_2_ (100 *μ*L) was added. Clotting times were recorded. Meanwhile, in PT assays, serums (50 *μ*L) mixed with 25 *μ*L of samples were incubated for 3 min at 37°C. PT assay reagent (50 *μ*L), which has been hatched for 10 min at 37°C, was then added and clotting time was recorded. Determination of TT and FIB was performed according to the manufacturer's recommendations (Shanghai Sun Biotech Co., Ltd., China). In the above tests, control solvent was used as control group, and breviscapine and vitamin K_1_ were used as positive control group. PT, APTT, TT, and FIB assays were conducted by Semiautomated Coagulation Analyzer.

### 2.6. Experimental Model and Drug Administration

Rats were randomly divided into seven groups with eight animals in each, whose gender was equally distributed throughout groups. Group 1 was the control and Group 2 was the model, and rats were given control solvent. Group 3 was model + aspirin (ASP), and rats were given 100 mg/kg ASP. Groups 4–7 were model + drugs, and rats were given 1.2 g/kg RPR extract. All treatments were performed by gavage and were administered seven times with an interval of 12 h.

After the fifth administration, model rats groups were placed in ice-cold water during the interval between two injections of adrenaline hydrochloride (Adr) to establish blood stasis model. All other rats were subcutaneously injected with Adr (0.8 mg/kg), except for the control rats, which were injected with 0.9% (w/v) NaCl saline solution. After 2 h, the rats were kept in ice-cold water (0–2°C) for 5 min and then reinjected with Adr (0.8 mg/kg) subcutaneously 2 h after the ice-bath to obtain blood stasis model. Rats were fasted overnight and administration continued after performing the model. Blood samples were collected 30 min after the last administration on the following day [8].

Compounds' experiment was similar to the above method. Group 1 and Group 2 were the same as the above. Group 3 was model + xiangdan injection (Xdi) (3.6 mL/Kg), Groups 4–7 were model + drugs, and rats were given 5 mg/kg compounds. All treatments were performed by caudal vein and administered seven times with an interval of 24 h.

After the sixth administration, the model rats with blood stasis were established by the above method.

### 2.7. Collection of Blood Sample

Rats were anesthetized with chloral hydrate (300 mg/kg) 18 h after the last injection of Adr, and blood was drawn from the abdominal aortas. Blood was collected using 8^#^ disposable vein infusion needles. 1 mL of blood was taken into centrifuge tube on standing position until the serum was fully precipitated. The serum was used to determine eNOS, ET-1, 6-Keto-PGF1*α*, and TXB_2_. Another part of the blood was collected into vacuum tube containing heparin to detect WBV, PV, PT, TT, APTT, FIB, ESR, and PCV, respectively.

### 2.8. Statistical Analysis

All experimental results were expressed as mean ± standard deviation (SD). Statistical analysis was performed with the SPSS19.0 software. Comparison between any two groups was evaluated using one-way analysis of variance (ANOVA). All graphics were drawn by GraphPad Prism 5.

## 3. Results

### 3.1. Effects on Plasma Coagulation Parameters In Vitro

**Figure 1:**
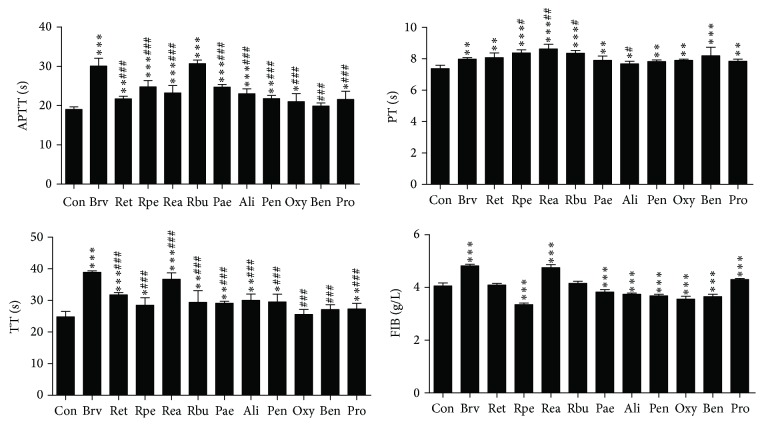
Anticoagulation activity of RPR extract and six compounds in vitro (*n* = 4; ^*∗∗∗*^*p* < 0.001 or 0.001 < ^*∗∗*^*p* < 0.01 or ^*∗*^*p* < 0.05 versus control group; ^###^*p* < 0.001 or 0.001 < ^##^*p* < 0.01 or ^#^*p* < 0.05 versus breviscapine).

As shown in Figure 1, compared with the control group, ethanol extract of RPR (Ret), petroleum extract of RPR (Rpe), ethyl acetate extract of RPR (Rea),* n*-butyl alcohol extract of RPR (Rbu), Pae, Ali, Pen, Oxy, and Pro could significantly prolong APTT (0.01 < *p* < 0.05; 0.001 < *p* < 0.01; *p* < 0.001, respectively). Above all, there was no difference between Rbu and breviscapine (Brv) (*p* > 0.05). In PT assay, compared with the control group, all of the extracts and six compounds could significantly prolong PT (0.01 < *p* < 0.05; 0.001 < *p* < 0.01; *p* < 0.001, respectively). Then the prolonging PT effects of Rpe, Rea, and Rbu were better than that of Brv (0.001 < *p* < 0.01; *p* < 0.001; resp.) and Ret, Pae, Pen, Oxy, Ben, and Pro had no difference with the Brv (*p* > 0.05). In TT assay, compared with the control group, the Ret, Rpe, Rea, Rbu, Pae, Ali, Pen, and Pro could significantly prolong TT (*p* < 0.001; 0.001 < *p* < 0.01; *p* < 0.05, resp.). For FIB assay, the Rpe, Pae, Ali, Pen, Oxy, and Ben could significantly decrease the content of FIB (*p* < 0.001), while the Ret, Rea, Rbu, and Pro could significantly increase the content of FIB (*p* < 0.001).

### 3.2. The Antithrombotic Effect of RPR Extract In Vivo

#### 3.2.1. Effects on WBV, PV, ESR, and PCV In Vivo

The effects on WBV and PV were shown in Table 1. In model group, WBV and PV significantly increased at all shear rates in the blood stasis (*p* < 0.001), which indicated the blood stasis rat model was successfully established. The Ret, Rea, and Rbu groups could significantly decrease WBV and PV at all shear rates (*p* < 0.001) and they decreased WBV and PV effects as well as aspirin group (*p* > 0.05).

The results of ESR and PCV were shown in Table 2. ESR and PCV in the model group were higher than that of control group (*p* < 0.001), which indicated that the blood stasis model was successfully established. The Ret, Rea, and Rbu groups could significantly decrease ESR and PCV (*p* < 0.001), and they decreased ESR effects as well as aspirin group (*p* > 0.05). The Rea and Rbu decreased PCV effects as well as aspirin group (*p* > 0.05).

#### 3.2.2. Effects on Plasma Coagulation Parameters

**Figure 2:**
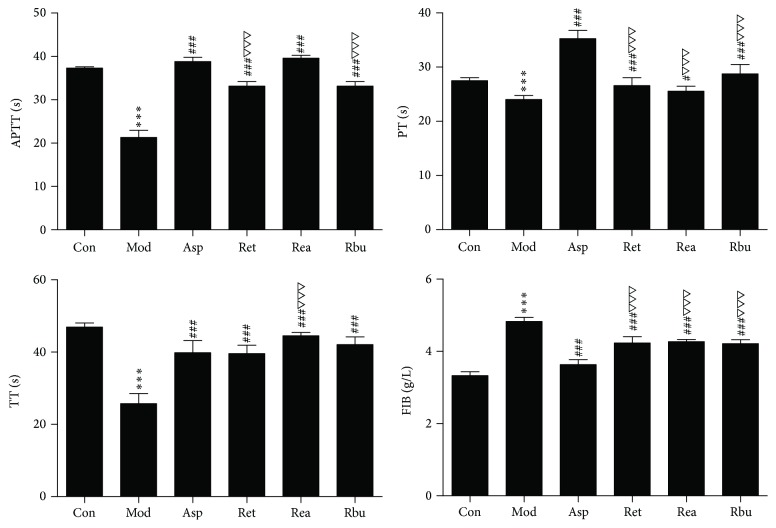
Anticoagulation activity of RPR extract in vivo (*n* = 8; ^*∗∗∗*^*p* < 0.001 versus control group; ^###^*p* < 0.001 versus model group; ^△△△^*p* < 0.001 versus aspirin; ^#^*p* < 0.05 versus model group).

To evaluate the effect of the coagulation cascade on the antithrombotic activity of RPR extract, the classical coagulation assays (APTT, TT, PT, and FIB content) were determined in vivo. Coagulation parameters were shown in Figure 2. APTT, TT, and PT were decreased, and FIB content was increased significantly in model group compared with control group (*p* < 0.001). RPR extracts could prolong PT, APTT, and TT and decrease FIB content significantly (*p* < 0.001).

#### 3.2.3. TXB_2_ and 6-Keto-PGF_1*α*_ Assay in Serum

Results of TXB_2_ and 6-Keto-PGF1*α* were shown in Table 3, and the plasma TXB_2_ level in model rats was prominently higher than that of control group (*p* < 0.001), whereas the plasma 6-Keto-PGF_1*α*_ level in model rats was markedly lower than that of control group (*p* < 0.05). Ret, Rea, and Rbu could significantly counteract increase of TXB_2_ level (*p* < 0.001) and the effects of Ret and Rea were close to aspirin (*p* > 0.05). Ret and Rbu could significantly upregulate 6-Keto-PGF1*α* level in plasma compared with model rats (*p* < 0.001, *p* < 0.05), and there were no obvious differences with aspirin (*p* > 0.05). Meanwhile, Ret, Rea, and Rbu decreased the ratio of TXB_2_/6-Keto-PGF_1*α*_ compared with model group (*p* < 0.001). The ratios of TXB_2_/6-Keto-PGF_1*α*_ in Ret was lower than that of aspirin (*p* < 0.001) and that of Rea was close to those of aspirin (*p* > 0.05).

#### 3.2.4. Effect of RPR on ET and eNOS

**Figure 3:**
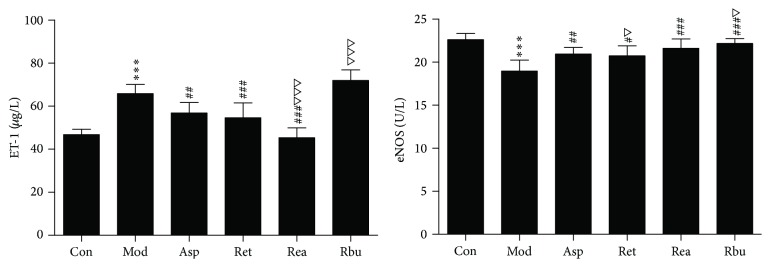
The effects of RPR treatment on eNOS and ET-1 level in thrombosis rats (*n* = 8; ^*∗∗∗*^*p* < 0.001 versus control group; ^###^*p* < 0.001 or 0.001 < ^##^*p* < 0.01 versus model group; ^△△△^*p* < 0.001 or ^△^*p* < 0.05 versus aspirin; ^#^*p* < 0.05).

In Figure 3, the plasma ET-1 level in blood stasis model rats (65.90 ± 4.24 *μ*g/L) was prominently higher than that of control group (46.75 ± 2.54 *μ*g/L) (*p* < 0.001), whereas the plasma eNOS level in model rats (18.96 ± 1.27 U/L) was markedly lower than that of control group (22.61 ± 0.73 U/L) (*p* < 0.001). Ret and Rea could effectively downregulate of ET-1 level and upregulate eNOS level in plasma compared with model rats (*p* < 0.001, *p* < 0.05, resp.), and Rea downregulation of ET-1 level effect was better than that of aspirin (*p* < 0.001), and the effect of Ret downregulation of ET-1 level had no obvious difference compared to aspirin group (*p* > 0.05). Ret, Rea, and Rbu could upregulate eNOS level in plasma compared with model rats (*p* < 0.001, *p* < 0.05, resp.) and the effects of Rbu upregulation of eNOS level were better than that of aspirin (*p* < 0.05), the effects of Rea upregulation of eNOS level had no obvious difference compared to aspirin (*p* > 0.05).

### 3.3. The Antithrombotic Effect of Active Compounds In Vivo

#### 3.3.1. Effects on WBV, PV, ESR, and PCV

The effects on WBV and PV were shown in Table 4. In model group, WBV and PV significantly increased at all shear rates in the blood stasis (*p* < 0.001), which indicated the blood stasis rat model was successfully established. Pro, Ali, Pae, and Pen could significantly decrease WBV and PV at all shear rates (*p* < 0.001; 0.001 < *p* < 0.01; *p* < 0.05) and they decreased WBV effects as well as xiangdan injection (Xdi) (*p* > 0.05) at low shear rate.

The results of ESR and PCV for each group are shown in Table 5. ESR and PCV in the model group were higher than that of control group (*p* < 0.001). Pro, Ali, Pae, and Pen could significantly decrease ESR and PCV (*p* < 0.001; 0.001 < *p* < 0.01; *p* < 0.05). Effects on ESR and PCV of Ali were equal to Xdi (*p* > 0.05). Effect on ESR of Pro was equal to Xdi (*p* > 0.05). The effect on PCV of Pae was equal to Xdi (*p* > 0.05).

#### 3.3.2. Effects on Plasma Coagulation Parameters

**Figure 4:**
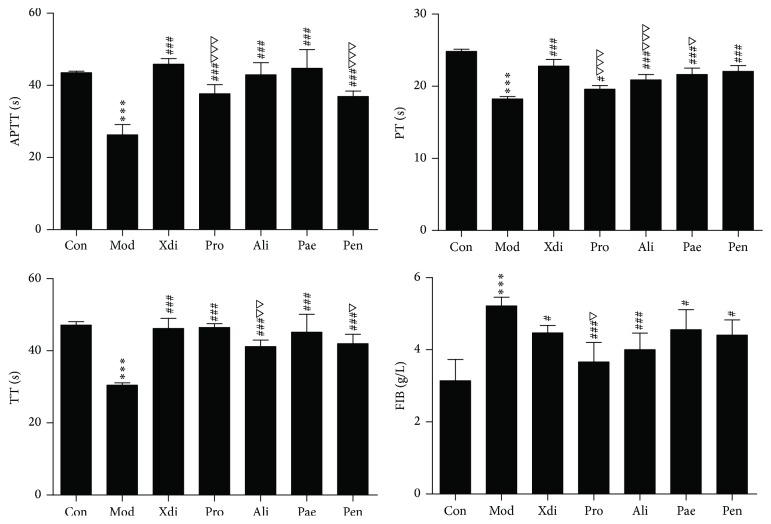
Anticoagulation activity of active compounds in vivo (*n* = 8; ^*∗∗∗*^*p* < 0.001 versus control group; ^###^*p* < 0.001 or ^#^*p* < 0.05 versus model group; ^△△△^*p* < 0.001 or ^△^*p* < 0.05 versus Xdi; 0.001 < ^△△^*p* < 0.01).

Coagulation parameters were shown in Figure 4. APTT, TT, and PT were decreased, and FIB content was increased very significantly in model group compared with control group (*p* < 0.001). Pro, Ali, Pae, and Pen could prolong PT, APTT, and TT and decrease FIB content effectively (*p* < 0.001, *p* < 0.05).

#### 3.3.3. TXB_2_ and 6-Keto- PGF_1*α*_ in Serum

In Table 6, the plasma TXB_2_ level in blood stasis model was prominently higher than that of control group (*p* < 0.001), whereas the plasma 6-Keto-PGF_1*α*_ level in model group was markedly lower than that of control group (*p* < 0.001).

Ali and Pen could significantly counteract increase of TXB_2_ level (*p* < 0.001) and they were close to those of Xdi group (*p* > 0.05). Pro, Ali, Pae, and Pen could effectively upregulate 6-Keto-PGF_1*α*_ level in plasma compared with model rats (*p* < 0.001, 0.001 < *p* < 0.01), and Pae and Pen had no obvious difference compared to Xdi (*p* > 0.05). Meanwhile, Pro, Ali, Pae, and Pen could decrease the ratio of TXB_2_/6-Keto-PGF_1*α*_ compared with model group (*p* < 0.001). Pro and Ali ratios of TXB_2_/6-Keto-PGF_1*α*_ in the rat were lower compared to the Xdi (*p* < 0.001) and those of the Pae and Pen were close to those of Xdi (*p* > 0.05).

#### 3.3.4. Effect of Compounds on ET and eNOS

**Figure 5:**
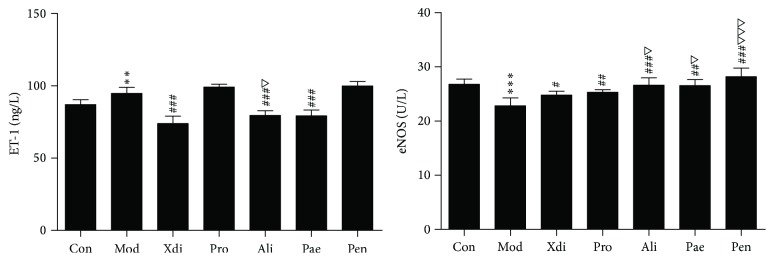
The effects of active compounds treatment on eNOS and ET-1 level in thrombosis rats (*n* = 8; ^*∗∗∗*^*p* < 0.001 versus control group; ^###^*p* < 0.001 or 0.001 < ^##^*p* < 0.01 versus model group; ^△△△^*p* < 0.001 or ^△^*p* < 0.05 versus Xdi; 0.001 < ^*∗∗*^*p* < 0.01 versus control group).

As shown in Figure 5, the plasma ET-1 level in blood stasis model rats (94.82 ± 4.19 *μ*g/L) was prominently higher than that of control group (87.08 ± 3.34 *μ*g/L) (*p* < 0.001), whereas the plasma eNOS level in model rats (22.83 ± 1.45 U/L) was markedly lower than that of control group (26.77 ± 0.98 U/L) (*p* < 0.001). Ali and Pae could effectively downregulate ET-1 level and upregulate eNOS level in plasma compared with model rats (*p* < 0.001, 0.001 < *p* < 0.01), and the Pae group downregulation of ET-1 level effect had no obvious difference compared to Xdi group (*p* > 0.05). Pro, Ali, Pae, and Pen could upregulate eNOS level in plasma compared with model rats (*p* < 0.001; 0.001 < *p* < 0.01; *p* < 0.05) and Ali and Pen upregulation of eNOS level effect was better than aspirin group (*p* < 0.001, *p* < 0.05).

## 4. Discussion

APTT, PT, TT, and FIB are four indicators, related to anticoagulant in clinical practice. In our study, Rpe, Pae, Ali, and Pen could significantly extend TT, APTT, and TT and reduced the content of FIB in vitro, which indicated the beneficial effect of them on intrinsic and extrinsic coagulation pathways, and hinder fibrin formation. Ret, Rea, Rbu, and Pro could significantly extend TT, APTT, and PT, but they could not reduce the content of FIB in vitro, which indicated the beneficial effect of them on intrinsic and extrinsic coagulation pathways. Then the Oxy and Ben had no anticoagulation effect in vitro.

Hemorheology is related to blood flow and pressure, flow volume, and resistance of blood vessels, including WBV, PV, ESR, and PCV. Hemorheology is commonly used for diagnosis and prevention of cardiovascular diseases in clinical practice [12–19]. Red blood cells (RBCs), platelets, and plasma have an influence on WBV, because RBCs account for almost 50% of blood volume and constitute the majority of the cellular content in blood. In our research, Ret, Rea, Rbu, Pro, Ali, Pae, and Pen groups could significantly decrease WBV and PCV which suggested that the amelioration effect of compounds on WBV might be partly due to the PCV decrease.

PV plays an important role in WBV. It showed the coefficient between PV and ESR was positive in significant level [20]. In our research, Ret, Rea, Rbu, Pro, Ali, Pae, and Pen groups could significantly decrease PV and ESR which suggested that the amelioration effect of compounds on PV might be partly due to the ESR decrease.

Ret, Rea, Rbu, Pro, Ali, Pae, and Pen groups could significantly extend TT, APTT, and PT and reduced the content of FIB in vivo, which indicated the beneficial effect of them on intrinsic and extrinsic coagulation pathways, and hinder fibrin formation. However, further study will be required to clarify these additional mechanisms.

TXA_2_ and PGI_2_, metabolites of arachidonic acid (AA), which are mutually antagonistic, play an important role in thrombosis. TXB_2_ and 6-Keto-PGF_1*α*_ are the stable metabolites of TXA_2_ and PGI_2_, respectively [21]. The dynamic equilibrium of TXB_2_/6-Keto-PGF_1*α*_ is a vital factor for the regulation and control of vessel wall intensity and regional flow [21, 22].

Our study results suggested that Ret, Rbu, Ali, and Pen could significantly downregulate serum TXB_2_ level and upregulate serum 6-Keto-PGF_1*α*_ level in blood stasis rat, which indicated that the antithrombotic effects of them were associated with the regulation of TXA_2_ and PGI_2_. Rea could significantly downregulate serum TXB_2_ level, which indicated that the antithrombotic effect of Rea was associated with the regulation of TXA_2_. Pro and Pen could significantly upregulate serum 6-Keto-PGF1*α* level, indicating that the antithrombotic action of Pro and Pen was associated with the regulation of PGI_2_.

Endothelial dysfunction plays a pivotal role in thrombosis [23, 24]. ET and NO, a pair of mutually antagonistic biological activity factor, play an important role in regulating cardiovascular system function and participate in a variety of pathological processes of cardiovascular disease. The damage of vascular endothelial cells increases the release of ET and leads to vasoconstriction [25]. NO synthase can reflect the changes in endogenous NO, a vasodilatory factor secreted by endothelial cells. ENOS, a constitutively present enzyme in distinct subcellular locations within cardiomyocytes, is mainly responsible for the endothelium-dependent vasodilatation distributed in vascular endothelium. And the activation of eNOS promotes the continued release of NO [26, 27]. A research [28] reported that RPR could relaxe vascular smooth muscle via endothelium-dependent and Akt- and SOCE-eNOS-cGMP-mediated pathways through activation of both K_Ca_ and K_ATP_ channels and inhibition of L-type Ca^2+^ channels. In our research, Rbu, Pro, and Pen could significantly increase eNOS level. Then Ret, Rea, Ali, and Pae could significantly decrease ET level and markedly increase eNOS level. These results indicated that Ret, Rea, Ali, and Pae had a protective effect on vascular endothelium and could regulate the tension of vascular smooth muscle by downregulating ET level and upregulating eNOS level.

In addition, Wang et al. [29] investigated water extract of RPR against myocardial ischemia in mice induced by isoproterenol and found that RPR has the cardioprotective activities on isoproterenol-induced myocardial ischemia. Myocardial ischemia is associated with myocardial infarction and sudden death; hence, antithrombotic effects of RPR may contribute to the improvement of myocardial ischemia. The research [30] reported that Pae has many pharmacological activities, such as protection function of the blood brain barrier, cerebral blood flow, brain edema after cerebral ischemia, antitumour effect, immune regulation, and hypoglycemic effect. Another research [31] showed that Pae and Ali could increase the numbers of white blood cells and reversed the atrophy of thymus. However cardiovascular research on Pen and Pro was little. In our study, Pro, Ali, Pae, and Pen were four antithrombotic components of RPR.

## 5. Conclusion

The present study suggested that the RPR extract and the Pro, Ali, Pae, and Pen possessed remarkable antithrombotic property in blood stasis model rats induced by ice water bath and subcutaneous injection of norepinephrine. This property could be associated with activating blood flow, anticoagulation activity, the regulation of active substances in vascular endothelium, and maintaining the balance of TXA_2_ and PGI_2_. In the end, the Pro, Ali, Pae, and Pen may be the main antithrombotic ingredient of RPR.

## Figures and Tables

**Table 1:** Effect on WBV (mPa·s) at various shear rates.

Group	WBV (mPa·s)			PV (mPa·s)
	200/S	30/S	3/S	30/s
Con	4.34 ± 0.20	5.45 ± 0.33	9.88 ± 0.61	1.23 ± 0.03
Mod	5.44 ± 0.17^*∗∗∗*^	6.80 ± 0.14^*∗∗∗*^	12.66 ± 0.25^*∗∗∗*^	1.42 ± 0.03^*∗∗∗*^
Asp	4.67 ± 0.47^###^	5.75 ± 0.19^###^	9.98 ± 0.36^###^	1.27 ± 0.03^###^
Ret	4.68 ± 0.17^###^	6.04 ± 0.45^##^	10.39 ± 0.68^###^	1.29 ± 0.01^###^
Rea	4.67 ± 0.46^###^	5.80 ± 0.56^###^	10.17 ± 0.41^###^	1.29 ± 0.05^###^
Rbu	4.64 ± 0.43^###^	5.60 ± 0.61^###^	10.38 ± 0.54^###^	1.26 ± 0.04^###^

Data represent mean ± SD. *n* = 8, ^*∗∗∗*^*p* < 0.001 versus control group, ^###^*p* < 0.001 and 0.001 < ^##^*p* < 0.01 versus model group.

**Table 2:** Effect on ESR and PCV.

Group	Dose (g/kg)	ESR (mm·h^−1^)	PCV (%)
Con	—	3.00 ± 0.08	37.15 ± 2.46
Mod	—	11.60 ± 1.14^*∗∗∗*^	46.83 ± 0.71^*∗∗∗*^
Asp	0.1	7.40 ± 0.55^###^	38.92 ± 1.04^###^
Ret	1.2	6.79 ± 0.84^###^	41.71 ± 1.30^###△^
Rea	1.2	7.80 ± 0.84^###^	39.73 ± 2.33^###^
Rbu	1.2	6.80 ± 0.84^###^	39.73 ± 1.82^###^

Data represent mean ± SD. *n* = 8, ^*∗∗∗*^*p* < 0.001 versus control group, ^###^*p* < 0.001 versus model group, and ^△^*p* < 0.05 versus aspirin.

**Table 3:** Effect on TXB_2_ and 6-Keto-PGF_1*α*_ in serum.

Group	Dose (g/kg)	6-Keto-PGF1*α* (ng/L)	TXB_2_	TXB_2_/6-Keto-PGF1*α*
Con	—	91.79 ± 7.38	83.36 ± 6.75	0.911 ± 0.08
Mod	—	81.26 ± 2.05^*∗*^	108.78 ± 3.79^*∗∗∗*^	1.34 ± 0.06^*∗∗∗*^
Asp	0.1	90.58 ± 4.56^#^	81.90 ± 4.38^###^	0.910 ± 0.07^###^
Ret	1.2	96.27 ± 8.13^###^	83.52 ± 3.74^###^	0.874 ± 0.10^###△△△^
Rea	1.2	86.81 ± 6.10	84.42 ± 7.96^###^	0.976 ± 0.11^###^
Rbu	1.2	91.24 ± 5.36^#^	99.24 ± 6.93^##△△△^	1.09 ± 0.09^###△△△^

Data represent mean ± SD. *n* = 8; ^*∗∗∗*^*p* < 0.001 or ^*∗*^*p* < 0.05 versus control group; ^###^*p* < 0.001 or 0.01 < ^##^*p* < 0.001 or ^#^*p* < 0.05 versus model group; ^△△△^*p* < 0.001 versus aspirin.

**Table 4:** Effect on WBV (mPa·s) at various shear rates.

Group	Dose	WBV (mPa·s)			PV (mPa·s)
		200/S	30/S	3/S	30/S
Con	—	3.96 ± 0.11	5.26 ± 0.09	10.06 ± 0.21	1.27 ± 0.04
Mod	—	5.29 ± 0.18^*∗∗∗*^	6.60 ± 0.23^*∗∗∗*^	12.55 ± 0.43^*∗∗∗*^	1.41 ± 0.03^*∗∗∗*^
Xdi	3.6 mL/Kg	4.14 ± 0.04^###^	5.28 ± 0.19^###^	10.49 ± 0.86^###^	1.27 ± 0.01^###^
Pro	5.0 mg/Kg	4.77 ± 0.09^###△△△^	5.82 ± 0.11^###△△△^	11.10 ± 0.18^###^	1.36 ± 0.03^#△△△^
Ali	5.0 mg/Kg	4.37 ± 0.17^###△△^	5.39 ± 0.26^###^	10.27 ± 0.47^###^	1.33 ± 0.03^###△^
Pae	5.0 mg/Kg	4.46 ± 0.11^###△△△^	5.58 ± 0.12^###△△^	10.62 ± 0.23^###^	1.35 ± 0.03^#△△△^
Pen	5.0 mg/Kg	4.63 ± 0.05^###△△△^	5.69 ± 0.99^###△△^	10.70 ± 0.39^###^	1.34 ± 0.02^##△△^

Data represent mean ± SD. *n* = 8, ^*∗∗∗*^*p* < 0.001 versus control group; ^###^*p* < 0.001, 0.001 < ^##^*p* < 0.01, and ^#^*p* < 0.05 versus model group; ^△△△^*p* < 0.001, 0.001 < ^△△^*p* < 0.01, and ^△^*p* < 0.05 versus Xdi.

**Table 5:** Effect on ESR and PCV.

Group	Dose	ESR (mm·h^−1^)	PCV (%)
Con	—	3.00 ± 0.82	36.04 ± 1.23
Mod	—	10.50 ± 0.58^*∗∗∗*^	45.80 ± 1.30^*∗∗∗*^
Xdi	3.6 mL/Kg	7.25 ± 0.50^###^	37.11 ± 1.73^###^
Pro	5 mg/Kg	8.50 ± 0.58^##^	40.73 ± 1.16^###△△^
Ali	5 mg/Kg	8.5 ± 1.29^##^	37.56 ± 2.37^###^
Pae	5 mg/Kg	8.75 ± 1.7^#△^	38.68 ± 1.48^###^
Pen	5 mg/Kg	8.90 ± 0.84^#△^	39.55 ± 1.48^###△^

Data represent mean ± SD. *n* = 8, ^*∗∗∗*^*p* < 0.001 versus control group, and ^###^*p* < 0.001 or 0.001 < ^##^*p* < 0.01 or ^#^*p* < 0.05 versus model group, and 0.001 < ^△△^*p* < 0.01 or ^△^*p* < 0.05 versus Xdi.

**Table 6:** Effect on TXB_2_ and 6-Keto-PGF_1*α*_ in serum.

Group	Dose	6-Keto-PGF1*α* (ng/L)	TXB_2_	TXB_2_/6-Keto-PGF1*α*
Con	—	1.358 ± 0.11	100.74 ± 2.58	1.358 ± 0.11
Mod	—	1.872 ± 0.10^*∗∗∗*^	109.63 ± 1.00^*∗∗∗*^	1.872 ± 0.10^*∗∗∗*^
Xdi	3.6 mL/Kg	1.178 ± .015^###^	100.24 ± 3.16^###^	1.178 ± .015^###^
Pro	5 mg/Kg	1.479 ± 0.04^###△△△^	106.25 ± 3.58	1.479 ± 0.04^###△△△^
Ali	5 mg/Kg	1.406 ± 0.11^###△△^	100.36 ± 1.05^###^	1.406 ± 0.11^###△△^
Pae	5 mg/Kg	1.208 ± 0.08^###^	106.42 ± 2.32	1.208 ± 0.08^###^
Pen	5 mg/Kg	1.198 ± 0.07^###^	101.23 ± 2.35^###^	1.198 ± 0.07^###^

Data represent mean ± SD. *n* = 8; ^*∗∗∗*^*p* < 0.001 versus control group; ^###^*p* < 0.001 versus model group; ^△△△^*p* < 0.001 or 0.01 < ^△△^*p* < 0.001 versus Xdi.

## References

[1] Berry C. N., Girard D., Lochot S., Lecoffre C. (1994). Antithrombotic actions of argatroban in rat models of venous, ‘mixed’ and arterial thrombosis, and its effects on the tail transection bleeding time. *British Journal of Pharmacology*.

[2] Bao Q. Q., Zhang L., Li X. Q., Li Z. L., Liu Y. M. (2009). Clinical significance of acute infection in elderly patients with thrombotic disease. *Chinese Journal of Gerontology*.

[3] Rosendaal F. R. (1999). Risk factors for venous thrombotic disease. *Thrombosis and Haemostasis*.

[4] Lu A.-P., Jia H.-W., Xiao C., Lu Q.-P. (2004). Theory of traditional chinese medicine and therapeutic method of diseases. *World Journal of Gastroenterology*.

[5] Xu S., Yang L., Tian R. (2009). Species differentiation and quality assessment of Radix Paeoniae Rubra (Chi-shao) by means of high-performance liquid chromatographic fingerprint. *Journal of Chromatography A*.

[6] Su J., Zhang P., Zhang J.-J., Qi X.-M., Wu Y.-G., Shen J.-J. (2010). Effects of total glucosides of paeony on oxidative stress in the kidney from diabetic rats. *Phytomedicine*.

[7] Zhang P., Zhang J.-J., Su J., Qi X.-M., Wu Y.-G., Shen J.-J. (2009). Effect of total glucosides of paeony on the expression of nephrin in the kidneys from diabetic rats. *The American Journal of Chinese Medicine*.

[8] Li H.-X., Han S.-Y., Wang X.-W. (2009). Effect of the carthamins yellow from *Carthamus tinctorius* L. on hemorheological disorders of blood stasis in rats. *Food and Chemical Toxicology*.

[9] Liu L., Duan J.-A., Tang Y. (2012). Taoren-Honghua herb pair and its main components promoting blood circulation through influencing on hemorheology, plasma coagulation and platelet aggregation. *Journal of Ethnopharmacology*.

[10] Gromotowicz A., Szemraj J., Stankiewicz A. (2011). Study of the mechanisms of aldosterone prothrombotic effect in rats. *JRAAS—Journal of the Renin-Angiotensin-Aldosterone System*.

[11] Chen H., Jin M., Wang Y.-F. (2014). Effect of *Toona microcarpa* harms leaf extract on the coagulation system. *BioMed Research International*.

[12] Gomi T., Ikeda T., Ikegami F. (1997). Beneficial effect of *α*-blocker on hemorheology in patients with essential hypertension. *Biorheology*.

[13] Koppensteiner R., Minar E., Ehringer H. (1990). Effect of lovastatin on hemorheology in type II hyperlipoproteinemia. *Atherosclerosis*.

[14] Hsieh C.-L., Lin Y.-C., Yen G.-C., Chen H.-Y. (2007). Preventive effects of guava (*Psidium guajava* L.) leaves and its active compounds against *α*-dicarbonyl compounds-induced blood coagulation. *Food Chemistry*.

[15] Elwan O., Al-Ashmawy S., El-Karaksy S., Hassan A. A. H. (1991). Hemorheology, stroke and the elderly. *Journal of the Neurological Sciences*.

[16] Dormandy J., Ernst E., Matrai A., Flute P. T. (1982). Hemorrheologic changes following acute myocardial infarction. *American Heart Journal*.

[17] Görög P., Ridler C. D., Rees G. M., Kovacs I. B. (1995). Evidence against hypercoagulability in coronary artery disease. *Thrombosis Research*.

[18] Wang J. R. (2005). Hemorrheology often relationship between measuring index and application value. *Chinese Journal of Hemorheology*.

[19] Folsom A. R., Wu K. K., Rosamond W. D., Sharrett A. R., Chambless L. E. (1997). Prospective study of hemostatic factors and incidence of coronary heart disease: the Atherosclerosis Risk in Communities (ARIC) Study. *Circulation*.

[20] Zhao Y. G., Xu Y., Wang L. (1997). Research related to Blood sedimentation and plasma viscosity. *Chinese Journal of Hemorheology*.

[21] Homo-Delarche F., Bach J. F., Dardenne M. (1988). In vitro inhibition of prostaglandin production by azathioprine and 6-merchaptopurine. *Prostaglandins*.

[22] Furuhashi N., Tsujiei M., Kimura H., Yajima A., Nagae H., Kimura C. (1991). Maternal and fetal atrial natriuretic peptide levels, maternal plasma renin activity, angiotensin II, prostacyclin and thromboxane A_2_ levels in normal and preeclamptic pregnancies. *Tohoku Journal of Experimental Medicine*.

[23] Cai H., Harrison D. G. (2000). Endothelial dysfunction in cardiovascular diseases: the role of oxidant stress. *Circulation Research*.

[24] Celermajer D. S., Sorensen K. E., Gooch V. M. (1992). Non-invasive detection of endothelial dysfunction in children and adults at risk of atherosclerosis. *The Lancet*.

[25] Haynes W. G., Webb D. J. (1994). Contribution of endogenous generation of endothelin-1 to basal vascular tone. *The Lancet*.

[26] Förstermann U., Münzel T. (2006). Endothelial nitric oxide synthase in vascular disease: from marvel to menace. *Circulation*.

[27] Huang P. L., Huang Z., Mashimo H. (1995). Hypertension in mice lacking the gene for endothelial nitric oxide synthase. *Nature*.

[28] Jin S. N., Wen J. F., Wang T. T., Kang D. G., Lee H. S., Cho K. W. (2012). Vasodilatory effects of ethanol extract of Radix Paeoniae Rubra and its mechanism of action in the rat aorta. *Journal of Ethnopharmacology*.

[29] Wang Z. B., Yang H., Guo C. Y. (2013). Protection of water extract from paeoniae radix rubra against myocardial ischemia in mice induced by isoproterenol. *Chinese Herbal Medicines*.

[30] Zheng S. C., Li X. Y., Ou Y. B., Sun R. (2012). Research development on pharmacological of paeoniflorin. *Chinese Journal of Pharmacovigilance*.

[31] Zhu Y., Wang L., Yang Z. (2016). Hematopoietic effects of paeoniflorin and albiflorin on radiotherapy-induced myelosuppression mice. *Evidence-Based Complementary and Alternative Medicine*.

